# A Paradigmatic Case of Genetic Overlap Between Neurodevelopment Disorders and Schizophrenia Aligning with the Neurodevelopmental Continuum Hypothesis

**DOI:** 10.3390/ijms26093970

**Published:** 2025-04-23

**Authors:** Federica Iannotta, Ilaria La Monica, Maria Rosaria Di Iorio, Vittorio Freda, Antonia Sica, Andrea de Bartolomeis, Lucio Pastore, Felice Iasevoli, Barbara Lombardo

**Affiliations:** 1 Section of Psychiatry, Department of Neuroscience, School of Medicine, University of Naples “Federico II”, Via Sergio Pansini 5, 80131 Naples, Italy; fede.iannotta.fi@gmail.com (F.I.); vittoriofreda35@gmail.com (V.F.); andrea.debartolomeis@unina.it (A.d.B.); felice.iasevoli@unina.it (F.I.); 2 CEINGE-Biotecnologie Avanzate Franco Salvatore, Via G. Salvatore 486, 80145 Naples, Italy; lamonica@ceinge.unina.it (I.L.M.); diiorio@ceinge.unina.it (M.R.D.I.); sicaa@ceinge.unina.it (A.S.); lucio.pastore@unina.it (L.P.); 3 Department of Molecular Medicine and Medical Biotechnologies, Federico II University, Via Sergio Pansini 5, 80131 Naples, Italy

**Keywords:** neurodevelopmental disorders, schizophrenia, intellectual disability, neurodevelopmental continuum, copy number variants

## Abstract

Schizophrenia (SCZ) is a complex mental disorder, whose pathogenesis involves both environmental and genetic factors. Genetic risk is conferred through a combination of common variants and rare mutations, with point mutations and copy number variants (CNVs). Many of the genetic variants associated with SCZ have pleiotropic effects, influencing brain development and being shared with other neurodevelopmental disorders (NDDs), such as intellectual disability (ID). This overlap supports the concept of a neurodevelopmental continuum, suggesting shared genetic risk, at least between SCZ and ID, and most presumably among SCZ and many other NDDs. Here, we describe the case of a male patient whose clinical features align with this hypothesis. He presented cognitive and behavioral impairments preceding psychotic symptoms, further reinforcing the genetic and clinical interaction between SCZ and other NDDs. The patient’s genetic profile was analyzed using array comparative genomic hybridization (a-CGH) and whole-exome sequencing (WES) to investigate the genetic determinants underlying his clinical condition. The genetic testing identified variants in loci associated with both SCZ and NDDs. Our findings highlight the need to integrate genetic assessments into psychiatrists’ clinical practice. Moreover, this report contributes to the current body of evidence supporting the thesis on the neurodevelopmental continuum of SCZ.

## 1. Introduction

Schizophrenia (SCZ) is a severe mental disorder, with a global incidence of approximately 1%. Its clinical features are highly variable, but are conventionally captured by three domains: positive, negative, and cognitive symptoms. Positive symptoms include delusions, hallucinations, formal thought disorders, and behavioral disorganization; negative symptoms are characterized by affective flattening, social withdrawal, anhedonia, and abulia. Finally, cognitive symptoms encompass deficits in attention, working memory, and executive functions [1,2].

SCZ shows high heritability and the characteristics of a polygenic disorder, with both common genetic variants and rare mutations contributing to SCZ risk. Genome-wide association studies (GWAS) have identified over a hundred common alleles associated with the risk of developing SCZ; however, these variants individually show a modest effect [3]. Copy number variants (CNVs) are structural variations in the genome involving deletions or duplications of DNA segments larger than 100 kilobases compared to the reference genome [4]: the presence of rare or ultra-rare CNVs has also been implicated in SCZ pathogenesis. These CNVs encompass genomic regions containing multiple genes, expressed in several cell types and tissues, and encoding for proteins involved in signaling pathways; they are often associated with a high risk of SCZ [5].

Many genes disrupted or duplicated by the presence of these CNVs exhibit pleiotropic effects; they have also been linked to the development of neurodevelopmental disorders (NDDs), such as autism spectrum disorder (ASD), intellectual disability (ID), and attention deficit hyperactivity disorder (ADHD) [6,7,8,9,10]. NDDs are characterized by early-onset alterations in brain development, although their symptoms may become evident later in life. These disorders often do not improve over time and their clinical manifestations may evolve throughout the patient’s lifespan. Recent studies suggest that SCZ and other NDDs not only share risk genes, but they are also influenced by similar genetic variants [11]. This overlap has led to the development of the concept of a “neurodevelopmental continuum”, where all NDDs, including SCZ, are seen as varying outcomes of early disruptions at different stages of brain development [12].

One notable genetic overlap is between SCZ and ID. ID is defined by early-onset impairments in cognitive abilities and adaptive behaviors, affecting the patient’s conceptual, social, and practical skills [13]. A genetic cause is suspected in most cases, particularly when no obvious environmental factors, such as complications during childbirth, are present. ID patients are typically diagnosed in their childhood; however, a significant number of patients may remain not properly diagnosed and genetically characterized, even during adulthood. Individuals with ID have a higher incidence of psychiatric comorbidities than the general population; for example, the incidence of SCZ in ID patients is estimated to be ten times higher than unaffected adults [14]. However, this figure is likely underestimated, due to the challenges in recognizing psychiatric symptoms in individuals with severe intellectual impairments. The observed genetic overlap between SCZ and ID underlines the need for refining the diagnostic approaches for patients affected by NDDs; their clinical features should be considered manifestations of complex pathogenesis, rather than strictly adhering to defined diagnostic boundaries.

Here, we describe the identification of several DNA variants in the context of a clinical behavioral disorder, relevant for refining the diagnosis and overall intervention beyond pharmacological treatment.

## 2. Case Presentation

### 2.1. Patient Report

**Figure 1:**
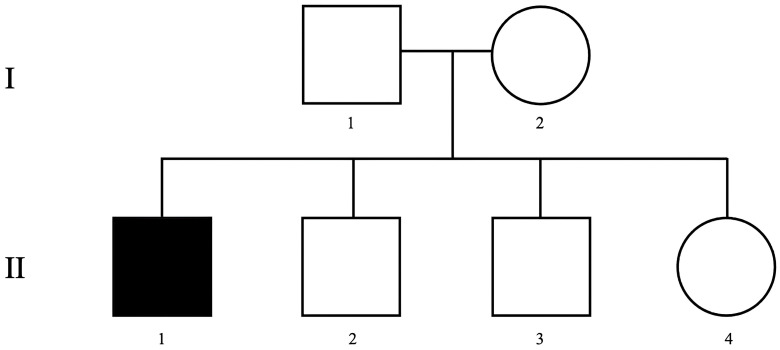
Pedigree of the studied family. Roman numbers indicate the different generations, while Arabic numbers represent the individuals within each generation.

AP is a 34-year-old male who was referred to the outpatient unit at the Hospital of the University of Naples Federico II because his case was initially considered as treatment-resistant psychosis, characterized by severe behavioral symptoms, unresponsive to antipsychotics. He manifested disorganized behavior, heightened excitement, grandiosity, and formal thought disorders, including loose associations of ideas and accelerated thought processes. The patient’s psychiatric and neurological history revealed two cousins with epilepsy and mood disorders, but no other significant familial medical history. He is the eldest of four children born to non-consanguineous parents via cesarean section (Figure 1).

Perinatal asphyxia necessitated 10 days of oxygen therapy, although this issue was resolved without sequelae. He experienced delays in acquiring motor and language skills, requiring speech therapy during childhood. Despite difficulties in school, he managed to complete his education and obtain a diploma. Despite early referral to child psychiatrists, no formal diagnosis of a psychiatric disorder was made at this age, mostly because the intensity and severity of the cognitive and behavioral manifestations were not considered severe enough to indicate a full-blown psychopathological condition. However, during adolescence, AP began exhibiting episodes of psychomotor agitation, disorganized behavior, megalomanic delusions, auditory hallucinations, and excessive, inappropriate spending. Additionally, he displayed repetitive, aggressive behavior and poor ideational content during periods of heightened stress. These behaviors prompted a new referral to psychiatric services and, ultimately, led to a diagnosis of SCZ; consequently, antipsychotic therapy was initiated. In adulthood, he was monitored by multiple psychiatric specialists. Although he occasionally held jobs, he struggled with excessive stress and faced persistent challenges in interpersonal relationships due to his incongruent behavior. He was described as overly friendly, naive, and prone to being deceived. As he did not respond adequately to antipsychotic treatment, he was considered a treatment-resistant patient and, so, was referred to our specialized unit for treatment-resistant psychosis.

At the time of evaluation at our clinic, the patient demonstrated clumsy movements, but appeared calm and cooperative. His parents reported ongoing aggressive behavior, behavioral disorganization, and delusional ideation, predominantly at home. In social settings, incongruous and bizarre behaviors were noted, such as poor sphincter control. His speech was repetitive and limited, with his thought contents revolving around a few basic concepts. Cognitive assessments revealed moderate-to-severe global impairment, depending on the task. At age 14, IQ testing indicated an IQ of 48. However, clinicians attributed this result to cognitive impairment due to early-onset SCZ rather than an ID. Despite early signs of NDD, no genetic etiology was suspected, and the patient was never referred for a genetic consultation. The patient was treated with a combination of lithium carbonate and paliperidone. Once symptom stabilization was achieved, he transitioned to monthly long-acting therapy. Nevertheless, he continued to require assistance with daily activities, such as shopping, washing, and cleaning. Because of his medical history and persistent symptoms, the patient was finally referred to genetic counseling. At the time of the medical genetic evaluation, several phenotypic abnormalities were observed, including narrow palpebral fissures, bilateral clinodactyly of the fourth fingers, flat feet, and bilateral rearfoot valgus. Based on these observations, array comparative genomic hybridization (a-CGH) analysis and whole-exome sequencing (WES) were performed. Table 1 provides the patient’s clinical and physical information at the first evaluation.

### 2.2. Molecular Analysis

After obtaining written informed consent, peripheral blood samples were collected from the proband and his parents for a-CGH and WES analyses. DNA was extracted from these samples using the Maxwell RSC Blood DNA Kit AS1400 (Promega, Madison, WI, USA). We performed the a-CGH analysis using a 2 × 400K GenetiSure Postnatal CGH+SNP Microarray (Agilent Technologies, Santa Clara, CA, USA), which provides an average spacing of approximately 9.5 kb and a resolution of 2.5 Mb for the copy-neutral loss of heterozygosity (LOH). Microarray scans were performed using an Agilent G2600D scanner, and the resulting image files were quantified using Agilent’s Cytogenomics software (v. 5.2.0.005). The human genome assembly GRCh38 (hg38) (https://www.ensembl.org/index.html, accessed on 17 July 2023) was used as a reference. CNVs identified in the interval-based report generated using Cytogenomics software were further analyzed using the Alissa bioinformatic software (Agilent, version 5.2.6). Databases including ClinVar (https://www.ncbi.nlm.nih.gov/clinvar/, accessed on 25 July 2023), Decipher (https://decipher.sanger.ac.uk/, accessed on 25 July 2023), the Database of Genomic Variants (http://dgv.tcag.ca/dgv/app/home, accessed on 25 July 2023), GeneCards (http://www.genecards.org/, accessed on 25 July 2023), and OMIM (https://www.omim.org/, accessed on 25 July 2023) were consulted for variant interpretation.

For the WES analysis, DNA libraries were prepared using the Human All Exon V7 targeted SureSelect XT HS enrichment system (Agilent Technologies, Santa Clara, CA, USA). Sequencing was performed on a Mid Output flow cell v. 2.5 (300 cycles), using a NextSeq 500 instrument (Illumina, San Diego, CA, USA). The resulting FASTQ files were imported into the seqr platform (seqr v. 1.0-d889cada) for sequence data analysis. Raw WES data from the proband are summarized in Table 2.

Approximately 14,000 variants were initially identified in the proband. We applied a stringent analysis pipeline using an in silico panel of genes specific to neurodevelopmental and psychiatric disorders. The variants were filtered based on population frequency, pathogenicity, and mutation annotations for effective prioritization. Prioritized variants were cross-referenced with the dbSNP (https://www.ncbi.nlm.nih.gov/snp/, accessed on 1 February 2024) and ClinVar databases. Their pathogenicity was further assessed using tools such as Varsome (https://varsome.com, accessed on 1 February 2024) and Franklin Genoox (https://franklin.genoox.com/clinical-db/home, accessed on 1 February 2024).

### 2.3. Results of Genetic Testing

**Figure 2:**
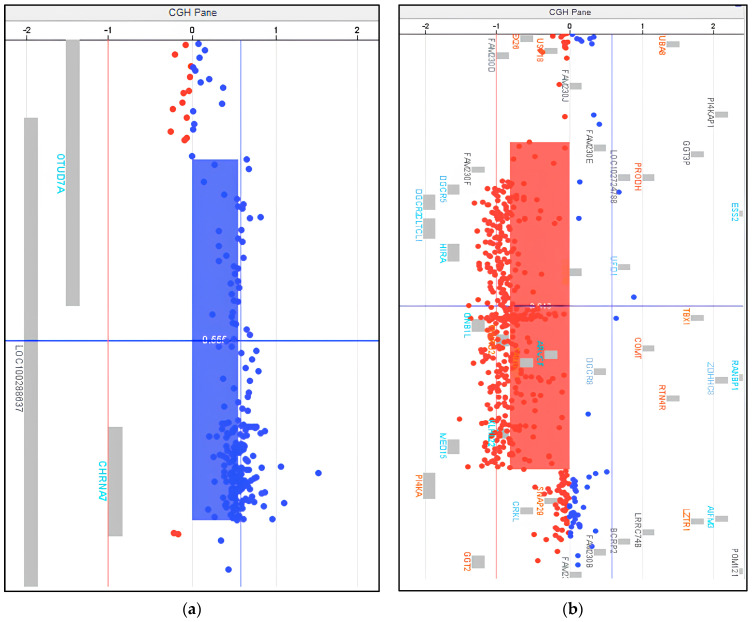
The a−CGH analysis results identified in the proband. (**a**) The a−CGH profile of chromosome 15. This analysis shows a heterozygous duplication of paternal origin in 15q13.3 of 472.6 Kb. (**b**) The a−CGH profile of chromosome 22. This analysis shows a heterozygous de novo deletion in 22q11.21 of 1967.4 Kb.

The a-CGH analysis revealed a paternally inherited duplication of uncertain clinical significance on chromosome 15, specifically in the q13.3 region (Figure 2a). This duplication spans 472.6 Kb, from nucleotide 31,680,443 to nucleotide 32,153,051, and partially involves the following genes: *CHRNA7* (cholinergic receptor nicotinic alpha 7), *OTUD7A* (OTU deubiquitinase 7), *ULK4P1* (ULK4 pseudogene 1), and *ULK4P2* (ULK4 pseudogene 2).

Most importantly, a de novo deletion of pathogenic clinical significance was identified in the 22q11.21 region. This deletion spans 1967.4 Kb, from nucleotide 18,718,488 to nucleotide 20,685,852 (Figure 2b), and fully involves several OMIM genes, including: *CDC45* (cell division cycle 45), *COMT* (catechol-O-methyltransferase), *DGCR2* (DiGeorge syndrome critical region gene 2), *DGCR8* (DiGeorge syndrome critical region gene 8), GP1BB (glycoprotein Ib platelet subunit beta), *GSC2* (goosecoid homeobox 2), *PRODH* (proline dehydrogenase 1), *RTN4R* (reticulon 4 receptor), *SCARF2* (scavenger receptor class F member 2), *SLC25A1* (solute carrier family 25 member 1), *TANGO2* (transport and Golgi organization 2 homolog), *TBX1* (T-box transcription factor 1), and *TXNRD2* (thioredoxin reductase 2).

In order to obtain a more detailed genetic characterization and a better understanding of the patient’s clinical presentation, WES was performed on the proband. Through the use of the prioritization process, nine variants potentially linked to the clinical phenotype were identified after filtering. The most relevant variants are listed in Table 3 and are discussed in detail in the following sections.

## 3. Discussion

In recent years, the combination of a-CGH and WES analyses has significantly contributed to the elucidation of the genetic etiology of several neurological disorders [15]; herein, these techniques provided a comprehensive analysis of the patient’s genetic alterations, helping to clarify his clinical presentation and identify potential variants linked to SCZ. The a-CGH analysis revealed a duplication of uncertain clinical significance on chromosome 15q13.3 and a clinically significant deletion on chromosome 22q11.21, both matching the patient’s phenotype. The 15q13.3 deletion is known to be associated with psychiatric disorders, often showing more severe effects than duplications. While deletions in this region are well-studied, the clinical significance of duplications, although rarer, remains less clear. A recent review found 241 deletions and 79 duplications in 15q13.3, with deletions typically linked to more severe phenotypes [16].

Chromosome 15q13.3 microduplications are associated with several neuropsychiatric conditions, including developmental delay, ID, epilepsy, and SCZ. The overlap region of these duplications contains the *CHRNA7* gene, which encodes for the α7 subunit of the nicotinic acetylcholine receptor (nAChR). Moreover, nAChRs are expressed in different brain areas, particularly in the hippocampus, a brain region important for cognitive processes. Through calcium signaling at the neuronal level, α7nAChRs are involved in regulating signal transmission, synaptic plasticity, learning, and memory [17]. Variations in *CHRNA7* dosage have been linked to NDDs, as evidenced by its inclusion in the Simons Foundation Autism Research Initiative database as a strong candidate gene, with a score of 2.1. Both deletions and duplications of *CHRNA7* have been associated with psychiatric symptoms like cognitive deficits, epilepsy, ASD, and SCZ, highlighting the importance of proper *CHRNA7* expression for normal brain function [18]. Variations in α7 expression may modify calcium signaling in the corresponding areas of the central nervous system [16]. In animal models, *CHRNA7* knockout mice showed compromised memory and attention, but no behavioral or neuropsychiatric changes were observed [15,19]. On the other hand, studies on induced pluripotent stem cells (iPSCs) and neural progenitor cells (NPCs) from patients with heterozygous deletions or duplications of the 15q13.3 locus revealed a shared mechanism that might be harmful, affecting *CHRNA7* dosage and reducing calcium flux, due to modifications in α7nAChR. In these models, the duplication led to increased *CHRNA7* expression, protein misfolding, and altered calcium flux, while the deletion reduced *CHRNA7* expression at the membrane, decreasing channel activity [20]. These findings emphasize the need for further research to fully understand how *CHRNA7* mutations impact neuropsychiatric phenotypes.

The deletion on chromosome 22q11.21 is a de novo mutation; this deletion has been well-described and causes 22q11.2 deletion syndrome (22q11.2DS), an autosomal dominant microdeletion with an estimated prevalence of 1:4000 live births [21]. More specifically, 22q11.2DS is linked to several physical and neuropsychiatric manifestations, including congenital heart defects, palate and throat abnormalities, immune dysfunction, and cognitive and psychiatric disorders, such as learning disabilities, anxiety, and SCZ. Individuals with 22q11.2DS often exhibit borderline intellectual function [22,23,24], with an increased incidence of psychiatric disorders, particularly SCZ [25,26,27]. Notably, 22q11.2DS is currently recognized as a genetic model for understanding the development of SCZ [28]. The genes in the 1.5 Mb critical region of 22q11.2 are key to understanding SCZ [29]. Genes in this region, such as *COMT* [30,31], *PRODH* [32,33], *DGCR2* [29], and *DGRG8* [34] are implicated in its pathogenesis. Also, the haploinsufficiency of some genes within the 22q11.2 region may contribute to the SCZ clinical phenotype, as 22q11 mRNA levels are reduced by 40–60% [35,36,37]. *COMT* encodes an enzyme that regulates dopamine in the prefrontal cortex (PFC), a region central to cognitive and psychiatric functions. A low-activity COMT variant (*158-Met*) is associated with SCZ, particularly due to its role in dopaminergic dysfunction in the PFC, a hallmark of SCZ [36,38]. *PRODH* is involved in glutamate and GABA neurotransmission, with proline accumulation linked to neurocognitive impairment. Mutations in *PRODH* lead to a disruption in sensorimotor gating in animal models, further supporting its role in SCZ [29,39,40]. Recent studies suggest a relationship between *PRODH* and *COMT*, where hyperprolinemia may exacerbate dopaminergic dysfunction, increasing the risk of psychosis, particularly in individuals with low COMT activity [41,42,43]. Additionally, *DGCR2*, another gene within the deleted region, is associated with SCZ through its role in dendritic spine development and cortical circuit regulation. Mouse models show that silencing *DGCR2* impairs corticogenesis and behavior, emphasizing its role in psychiatric phenotypes [44,45,46]. The *DGCR8* gene, involved in microRNA biogenesis, is also critical in 22q11.2DS. A hemizygous deficiency of *DGCR8* disrupts miRNA processing, leading to cognitive and behavioral deficits, as observed in mice [47,48]. The dysregulation of specific miRNAs in the PFC may underlie SCZ-related phenotypes in this population [49].

Among the genetic variants identified by WES analysis, lysine demethylase 5B (*KDM5B*) has garnered attention due to its role in NDDs. KDM5B encodes a lysine-specific histone demethylase, belonging to the family of Jumonji/ARID domain-containing histone demethylases [50]. This protein is upregulated in tumor cells, contributes to the transcriptional repression of tumor suppressor genes, and may play a role in DNA repair processes and genomic stability. A critical post-translational modification, histone H3 lysine 4 methylation (H3K4me), is associated with active gene transcription. Recent advances in next-generation sequencing (NGS) have linked mutations in H3K4me regulators to NDDs such as SCZ, ASD, and ID. Notably, loss-of-function mutations in histone demethylases like KDM5A, KDM5B, and KDM5C have been implicated in these conditions [51]. A study involving a *Drosophila melanogaster* model for ID and ASD demonstrated that reduced KDM5 expression led to intestinal barrier failure and changes in social behavior, correlated with shifts in gut microbiota composition. Moreover, KDM5 was found to transcriptionally regulate genes in the immunodeficiency (IMD) signaling pathway, which in turn helps maintain commensal bacterial homeostasis in a demethylase-dependent manner. This finding suggests that modifying the gut microbiome could be a potential therapeutic strategy for patients with ID and ASD [52].

Another gene implicated in NDDs is *SHANK2*, which encodes a member of the Shank protein family. This protein is involved in dendritic spine maturation and synaptic induction and is localized at the postsynaptic membrane of excitatory neurons [53]. SHANK2 functions as a molecular scaffolding in the postsynaptic density of excitatory synapses. Mutations in *SHANK2* have been linked to an increased risk of NDDs, such as ASD, and there is emerging evidence suggesting its involvement in the pathogenesis of SCZ. The functional impact of *SHANK2* mutations was previously assessed by overexpressing or knocking down *SHANK2* in primary hippocampal neuron cultures; the results revealed significant deficits in synaptic function, including reduced dendritic spine volume, altered synaptic clustering, and a loss of presynaptic contacts [54]. An additional study observed patients with de novo *SHANK2* deletions due to inherited CNV variations in the 15q11-q13 region, which contains the *CHRNA7* gene. This study supports the “multiple hit model” of ASD, which proposes that synaptic dysfunction, particularly involving *SHANK2* and *CHRNA7* mutations, contributes to the development of these NDDs [55].

## 4. Conclusions

The findings from the present case present two thought-provoking considerations that could serve as potential starting points for further research. The genetic architecture of NDDs, including SCZ, generally involves multiple factors: common genetic variants of modest effect, which contribute to polygenic risk scores; and rare or ultra-rare de novo mutations, such as CNVs or mutations in critical single genes. However, the case presented here may suggest a third possibility. Specifically, high susceptibility to NDDs could arise from the co-occurrence of inherited and de novo mutations in a limited set of high-risk genes. The cumulative effect of these mutations might fall below the threshold of a polygenic risk score that would typically indicate high susceptibility, but their combined neurobiological impact could still significantly impair typical neurodevelopment and contribute to the clinical phenotype.

Recent studies have highlighted the relationship between CNVs and single nucleotide polymorphisms (SNPs), revealing a reduced polygenic risk score in SCZ cases carrying CNVs strongly associated with the disorder. Notably, this effect was not observed in cases with CNVs of low-to-moderate effect on SCZ risk, which instead demonstrated an additive relationship with the polygenic risk score [55]. The case we present here exemplifies this complex interaction and underscores the need for more comprehensive genetic studies in NDDs. These studies should consider diverse genetic risk factors and explore all potential biological pathways influenced by the disrupted genes.

Moreover, the genetic boundaries between SCZ and other NDDs are becoming increasingly difficult to delineate. Many genetic lesions overlap across these conditions, which may explain both the clinical heterogeneity observed in SCZ and the frequent challenges in regard to distinguishing it from other NDDs. While these findings suggest the need for a re-evaluation of how we categorize and conceptualize SCZ, it may be more accurate to consider it as a “neurodevelopmental disorder with varying clinical manifestations”. Indeed, this case presents some limitations. Firstly, the study is based on a single case, limiting the possibility of generalizing our observations. Secondly, the pathogenic role of some genetic variants, such as the 15q13.3 duplication, remains debated. Although the literature supports their involvement in neurodevelopmental conditions, establishing direct causality in this case is still challenging, and more reports are needed to confirm our findings. Finally, due to the nature of this report, we are not able to provide a long-term clinical follow-up, limiting the understanding of the disease progression over time.

## Figures and Tables

**Table 1:** Patient’s characteristics at the first assessment, including the final therapy.

Clinical Characteristics	Details
Age at evaluation	32
Sex	Male
Referred diagnosis	Treatment- resistant schizophrenia
Onset	Childhood
Pharmacological treatment at evaluation	Valproic acid 1000 mg/day; aripiprazole 400 mg/monthly; fluvoxamine 100 mg/day; delorazepam 6 mg/day; levomepromazine 25 mg/day
Physical characteristics	Narrow palpebral fissures; fourth fingers clinodactyly; flat feet; rearfoot valgus (bilaterally)
PANSS score total	115
PANSS Positive score	22
PANSS Negative score	26
PANSS General score	67
IQ assessment	48
Current pharmacological treatment	Paliperidone monthly, lithium carbonate

**Table 2:** Raw data obtained by WES analysis.

Patient	Number of Reads	Average Depth	Coverage Percentage (≥50×)	Number of Variants (Total)
Proband (II.1)	31,477,808	193.36	91.2%	14,869

**Table 3:** Most relevant variants identified using the analysis pipeline.

Chr	Gene	Nucleotide Variant *	Protein *	Status	ACMG # Classification
1	*KDM5B*	NM_006618.5: c.3007T>A (exon 20)	p.Tyr1003Asn	Het	VUS
11	*SHANK2*	NM_012309.5: c.332G>A (exon 3)	p.Arg111His	Het	VUS

* According to the Human Genome Variation Society (HGVS) guidelines; # ACMG, America College of Medical Genetics; Het, heterozygous; VUS, variant of unknown significance.

## Data Availability

The data will be made available by contacting the corresponding authors.
